# Investigation of Invigorating *Qi* and Activating Blood Circulation Prescriptions in Treating *Qi* Deficiency and Blood Stasis Syndrome of Ischemic Stroke Patients: Study Protocol for a Randomized Controlled Trial

**DOI:** 10.3389/fphar.2020.00892

**Published:** 2020-06-17

**Authors:** Yu Wang, Ling Zhang, Yuan-jiang Pan, Wei Fu, Shu-wei Huang, Bin Xu, Li-ping Dou, Qun Hou, Chang Li, Li Yu, Hui-fen Zhou, Jie-hong Yang, Hai-tong Wan

**Affiliations:** ^1^Institute of Cardio-cerebrovascular Disease, Zhejiang Chinese Medical University, Hangzhou, China; ^2^Department of Chemistry, Zhejiang University, Hangzhou, China; ^3^Department of Cardiac-Cerebral Diseases, Yinchuan Cardiac-Cerebral Treatment Internet Hospital, Yinchuan, China; ^4^Department of Cardiovascular Diseases, The Second Affiliated Hospital of Zhejiang Chinese Medical University, Hangzhou, China; ^5^Department of Neurology, The Second Affiliated Hospital of Zhejiang Chinese Medical University, Hangzhou, China; ^6^Department of Neurology, The First Affiliated Hospital of Zhejiang Chinese Medical University, Hangzhou, China; ^7^Basic Medical and Public Health College, Zhejiang Chinese Medical University, Hangzhou, China

**Keywords:** traditional Chinese medicine, prescription–syndrome correlation, *Qi* deficiency and blood stasis syndrome, ischemic stroke, study protocol

## Abstract

Ischemic stroke (IS) is characterized by high morbidity and high mortality. The integration of Traditional Chinese medicine (TCM) and western medicine has shown promising benefits in relieving symptoms, promoting neurological recovery, and improving the quality of life of patients with IS. In TCM, *Qi*-deficiency along with blood-stasis (QDBS) syndrome is one of the common types of IS that is treated by invigorating *Qi* and activating blood circulation. In TCM theory, improving the corresponding degree of prescription–syndrome correlation (PSC) is helpful to improve clinical efficacy. In this study, we intend to use similar prescriptions that invigorate *Qi* and activate blood circulation: *Buyang Huanwu* granules (BHG), *Naoxintong* capsules (NXTC), and *Yangyin Tongnao* granules (YTG). The goal is to evaluate their level of PSC inpatients with IS with QDBS syndrome and find relevant biomarkers to provide an objective basis for precise treatment of TCM and improve the clinical therapeutic effects. A multicenter, randomized, double-blinded, and placebo-controlled intervention trial will be conducted in IS patients with QDBS syndrome, followed by an add-on of Chinese patent medicine. A total of 160 subjects will be randomly assigned to the BHG, NXTC, YTG, and placebo groups in a 1:2:1:1 allocation ratio. All subjects will undergo 28 days of treatment and then followed for another 180 days. The primary outcome is the changes in the National Institutes of Health Stroke Scale score after 28 days of medication. The secondary outcomes include the modified Rankin scale score, activity of daily living scale score, and TCM symptom score. Data will be analyzed in accordance with a predefined statistical analysis plan. Ethical approval of this trial has been granted by the Research Ethics Committee of the First Affiliated Hospital of Zhejiang Chinese Medical University (ID: 2017-Y-004-02). Written informed consent of patients will be required. This trial is registered in the Chinese Clinical Trial Registry (ChiCTR1800015189), and the results will be disseminated to the public through peer-reviewed journals and academic conferences.

## Introduction

Stroke is the second leading cause of death and a leading cause of disability worldwide (Feigin et al., 2014; Norberto et al., 2017). Ischemic stroke (IS) is the most common type and occurs after thrombosis in the brain or neck blood vessels (Fukui et al., 2017). From 1990 to 2010, the absolute number of first-ever IS patients increased by 37%, mortality increased by 21%, and the loss of disability-adjusted life-years increased by 18% in low- and middle-income countries (Feigin et al., 2014). One of the most important therapeutic strategies for IS is to promote neurological function recovery, and therefore, it is of great practical significance to search for therapeutic targets and drugs to improve neurological function in the later stages of IS. Traditional Chinese medicine (TCM) is a unique and complex medical system that developed over thousands of years and is widely used in Asia as a supplement therapy to western medicine in treating IS. The integration of TCM and western medicine has shown a promising benefit in relieving symptoms, promoting neurological recovery, and improving the quality of life of patients with IS (Wu et al., 2007; Chen et al., 2013; Zhang et al., 2013).

The concept of TCM syndromes (*zheng* in Chinese) reflects inherent pathological variations in the clinic; this important component of TCM theory contributes to identifying human body patterns and guides TCM treatments with herbs (Zhou et al., 2019). In TCM, “prescription–syndrome correlation” (PSC) refers to the high correlation between the compatibility of herbal medicines in a prescription and the disease mechanism that the prescription is directed against (Xin et al., 2010). Since the therapeutic effect is the sole criterion to judge the PSC, improving the corresponding degree of the PSC is the key way to enhance clinical efficacy and also the goal of TCM clinical practice and basic research (Lu et al., 2014). At present, the evaluation of clinical efficacy after TCM treatment is mainly based on improvement of the patients’ syndromes (symptoms, tongue, and pulse). However, due to the subjectivity and fuzziness of the syndrome concept, it is difficult to objectively evaluate the prescription, syndrome, and curative effect. For this reason, we will combine subjective symptoms with objective modern medical diagnosis indicators to objectively clarify the degree of correlation between prescription and syndrome.

*Qi*-deficiency with blood-stasis (QDBS) syndrome is a common type of IS addressed in TCM. Hemiplegia, hypoesthesia, white complexion, shortness of breath, fatigue, spontaneous sweating, dull dark and greasy or bitten tongue, and weak or irregular pulse are usually observed in patients with QDBS. Invigorating *Qi* and activating blood circulation (*Yiqi Huoxue* in Chinese) is the most effective IS treatment in TCM, and its corresponding representative prescription is *Buyang Huanwu* decoction (BHD) proposed by *Wang Qing-ren* in the *Qing* dynasty. BHD has been used to treat IS with QDBS syndrome for nearly 200 years, and it is now used as a basic prescription to treat QDBS in IS. A large number of studies have been carried out to assess the clinical efficacy, underlying mechanisms, and drug substance basis of BHD (Qu et al., 2014; Pan et al., 2017; Ma et al., 2018; Zhang et al., 2018). However, based on the theories of PSC, due to a weak ability of BHD in dredging collaterals, *Naoxintong* capsules (NXTC) was proposed as a supplement to BHD. NXTC is a prescription to invigorate *Qi*, activate blood circulation, ameliorate stasis, and enhance collateral dredging (‘Chinese Experts Consensus on Clinical Application of Naoxintong Capsule’ compilation team, 2017).

Based on previous clinical and basic studies and the theories of *Qi-Yin* related in TCM, overuse or long-term use of BHD may cause *Yin* injury and bleeding (Wan et al., 2015; Wang, 2018). Moreover, purely invigorating *Qi* and promoting blood circulation ignores the elderly pathogenesis of *Yin* deficiency on the treatment of IS with QDBS syndrome (Wan et al., 2015). Thus, *Yangyin Tongnao* granules (YTG) were developed as a prescription for nourishing *Yin*, invigorating *Qi*, and activating blood circulation.

In this study, we will use similar prescriptions for invigorating *Qi* and activating blood circulation (BHD, NXTC, and YTG; compositions listed in **Table 1**) to carry out clinical and biological research on PSC. We will evaluate the level of PSC for *Buyang Huanwu* granules (BHG), NXTC, and YTG in IS with QDBS syndrome, identify helpful clinical biomarkers that are related to IS with QDBS syndrome, and provide an objective basis for precise TCM treatment that can improve clinical therapeutic effect in patients with IS.

**Table 1:** Components of three prescriptions.

Prescription	*YiQi* herbs	*Huoxue* herbs	*Tongluo* herbs	*YangYin* herbs
**BHG**	*Astragalus mongholicus* Bunge (Radix Astragali)	*Angelica sinensis* (Oliv.) Diels (Angelicae Sinensis Radix), *Paeonia lactiflora* Pall. (Paeoniae Radix Rubra), *Oligochaeta*, Lumbricidae (Lumbricus rubellus), *Ligusticum chuanxiong* Hort. (Chuan xiong Rhizoma), *Carthamus tinctorius* L. (Carthami Flos), *Prunus persica* (L.) Batsch (Semen Persicae)	/	/
**NXTC**	*Astragalus mongholicus* Bunge (Radix Astragali)	*Angelica sinensis* (Oliv.) Diels (Angelicae Sinensis Radix), *Paeonia lactiflora* Pall. (Paeoniae Radix Rubra), *Ligusticum chuanxiong* Hort. (Chuanxiong Rhizoma), *Prunus persica* (L.) Batsch (Semen Persicae), *Carthamus tinctorius* L. (Carthami Flos), *Oligochaeta*, Lumbricidae (Lumbricus rubellus), *Hirudo nipponia* Whitman (Whitmania pigra Whitman), *Boswellia carterii* Birdw., (Boswellia carterii), *Commiphora myrrha Engl.*, (Myrrha), *Salvia miltiorrhiza* Bge. (Salviae Miltiorrhizae Radix Et Rhizoma)	*Achyranthes bidentata* Blume (Achyranthes), *Cinnamomum cassia* (L.) J. Presl (Cassia Twig), *Morus alba* L. (Mulberry Twig), *Spatholobus suberectus* Dunn (Caulis Spatholobi), *Buthus martensii* Karsch (Scorpion)	/
**YTG**	*Astragalus mongholicus* Bunge (Radix Astragali)	*Ligusticum chuanxiong* Hort. (Chuanxiong Rhizoma), *Hirudo nipponia* Whitman (Whitmania pigra Whitman)	/	*Dendrobium nobile* Lindl. (Dendrobium candidum), *Rehmannia glutinosa* (Gaertn.) DC. (Radix Rehmanniae), *Pueraria montana* var. lobata (Willd.) (Puerariae lobatae radix)

## Methods and Design

### Trial Design

This trial will be a multicenter, randomized, double-blinded, placebo-controlled intervention study of IS with QDBS syndrome, followed by an add-on of Chinese patent medicine. It is designed to evaluate the efficacy and safety of BHG, NXTC, and YTG for treating IS with QDBS syndrome. The patients will be randomly allocated to four parallel treatment groups using a 1:2:1:1 ratio.

**Figure 1:**
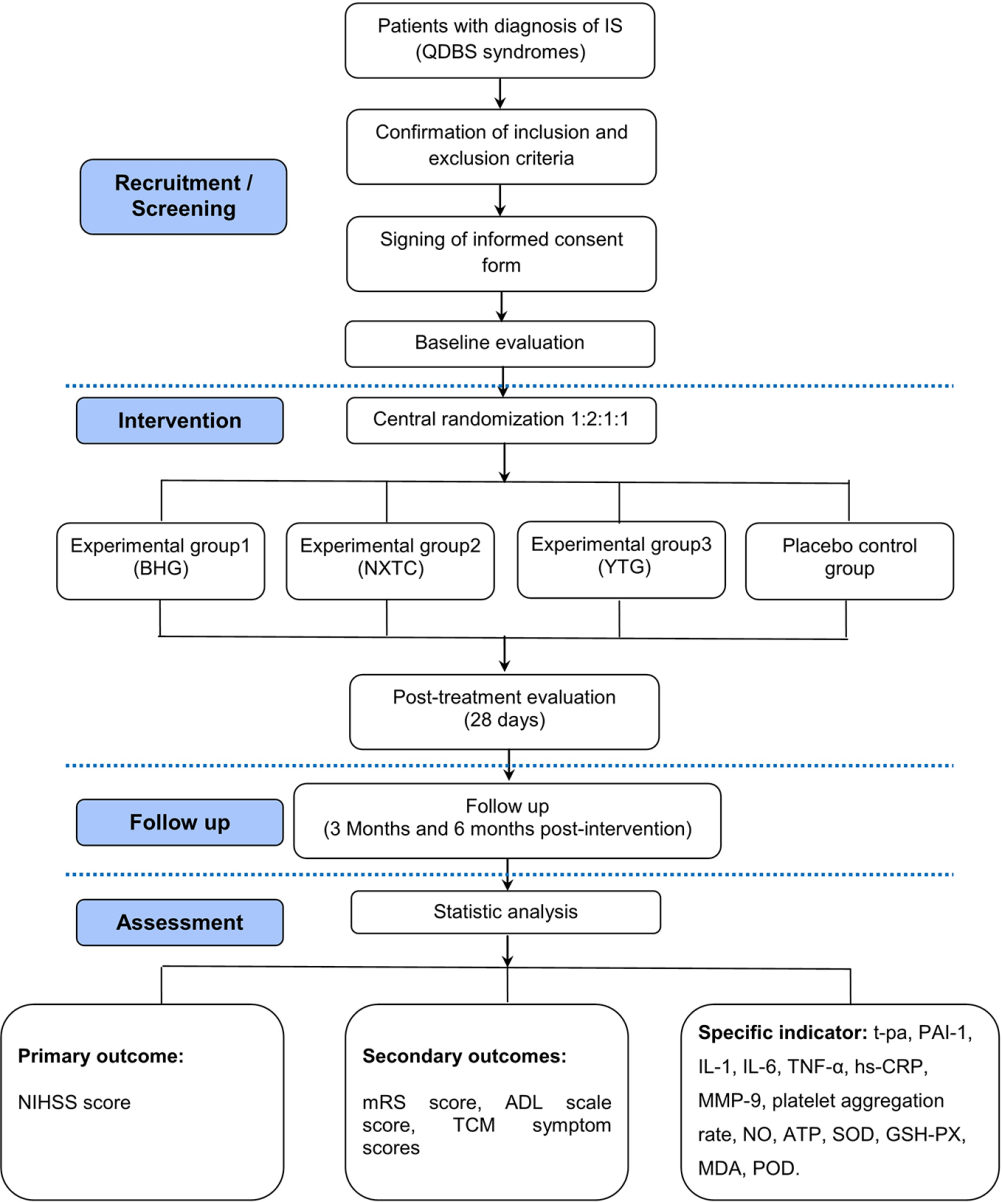
Flow chart of the study design.

This trial is registered in the Chinese Clinical Trial Registry (No. ChiCTR1800015189) and is in full compliance with the principles of the Declaration of Helsinki and Good Clinical Practice (GCP) guidelines. The protocol has been designed according to the following Standard Protocol Items: Recommendations for Interventional Trials (SPIRIT) checklist and the Recommendations for Interventional Trials and 2013 statement for herbal interventions (Chan et al., 2013a; Huang et al., 2019). We will rigorously follow the latest Consolidated Standards of Reporting Trials (CONSORT 2017) for Chinese herbal medicine recommendations (Cheng et al., 2017). The multicenter clinical trial will be conducted at seven hospitals in China (**Table 2**), and a total of 160 participants will be recruited. After the participants have provided informed consent, they will be enrolled in the trial, which consists of a 28-day treatment period and a 180-day follow-up period. The schematic diagram of study procedures is illustrated in **Figure 1**.

**Table 2:** Hospitals participating in this trial.

Code	Participating hospitals
01	Neurology Department, First Affiliated Hospital of Zhejiang Chinese Medical University
02	Neurology Department, Second Affiliated Hospital of Zhejiang Chinese Medical University
03	Encephalopathy Clinic, Hangzhou Xiaoshan District Hospital of Traditional Chinese Medicine
04	Neurology Department, Huai’an Second People’s Hospital
05	Neurology Department, Third People’s Hospital of Huzhou
06	Encephalopathy Clinic, Luohe Hospital of Chinese Medicine
07	Encephalopathy Clinic, Jinzhou Hospital of Chinese Medicine

### Ethics and Dissemination

The research team will protect the participants’ rights and safety by adherence to the Declaration of Helsinki, institutional policies and the International Conference on Harmonization-Good Clinical Practice (ICH-GCP). The study was approved by the Research Ethics Committee of the First Affiliated Hospital of Zhejiang Chinese Medical University (ID: 2017-Y-004-02). Eligible participants wishing to participate will be required to sign a written informed consent form. All information collected will be kept strictly confidential. Results of this randomized controlled trial (RCT) will be disseminated to the public through peer-reviewed journals and presentations at local, national, and international academic conferences.

### Diagnostic Criteria of IS

The western medicine diagnostic criteria of IS will refer to the ‘Guidelines for the diagnosis and treatment of acute ischemic stroke in China’ (2014 edition) (Chinese Medical Association, 2015) and the ‘Guidelines for the Treatment of Cerebro-vascular Diseases in China’ (2010 edition) (Chinese Medical Association, 2010). The Chinese medicine diagnostic criteria of IS will refer to the ‘Guiding Principles for Clinical Study of New Chinese Medicines’ (Ministry of Health of the People’s Republic of China, 2002).

### Diagnostic Criteria of TCM Syndrome

The diagnostic criteria of QDBS syndrome in TCM will refer to the ‘Guiding Principles for Clinical Study of New Chinese Medicines’ (Ministry of Health of the People’s Republic of China, 2002) and ‘Syndrome Element Differentiation’ (Zhu, 2008). Syndrome elements are the smallest diagnostic unit for the diagnosis of TCM syndromes (Wang et al., 2012). QDBS syndrome is the combination of “*Qi* deficiency,” “blood stasis,” and “mind” syndrome elements. TCM syndromes of each participant will be blindly evaluated by two Chinese medicine experts (Huang et al., 2019). Consistency will be checked with the kappa test.

### Inclusion Criteria

Patients will be recruited for this study if they meet all of the following criteria: (1) 40 ≤ age ≤ 80 years, (2) consistent with the western and Chinese medicine diagnostic criteria of IS, (3) meet the diagnostic criteria of TCM symptom differentiation (QDBS syndrome), (4) imaging (computed tomography or magnetic resonance imaging) confirmed anterior circulation infarction (Oxfordshire Community Stroke Project clinical classification of complete or partial anterior circulation infarction), (5) course of disease of 1–4 weeks, (6) 4 ≤ National Institute of Health Stroke Scale (NIHSS) score ≤ 20, (7) first attack or previous history of cerebral infarction but without disability before this attack (modified Rankin Scale [mRS] score ≤1), and (8) signed informed consent.

### Exclusion Criteria

The exclusion criteria will be as follows: (1) accompanied by cerebral hemorrhage tendency, cerebral hemorrhage after cerebral infarction, or had cerebral hemorrhage within 3 months; (2) combined with depression or dementia after stroke, posterior circulation infarct, transient ischemic attack, abnormal intracranial vascular networks, asymptomatic cerebral infarction, or large area of cerebral infarction with unstable vital signs; (3) subject withdrawal of TCM prescriptions for treating IS less than one week, or had used thrombolytic therapy after the onset of the disease; (4) patients with comorbidity or condition that would confound the neurological and functional evaluations; (5) clinical examination suggesting hemiplegia caused by brain tumors, brain trauma, or cerebral parasitic diseases, and cerebral embolism caused by rheumatic heart disease, coronary heart disease, and other heart diseases with atrial fibrillation; (6) patients unable to live independently or unsuitable for efficacy evaluation due to various diseases; (7) other serious cardio/cerebro-vascular, liver, kidney, hematopoietic system diseases and those who have undergone major surgery recently; (8) psychiatric patients, patients with severe depression, alcohol addicts or history of substance abuse; (9) females who are pregnant or lactating or have a positive pregnancy test at time of admission; (10) allergic to research drugs and its ingredients or have severe allergies; (11) participation in other clinical trials in the previous 3 months; or (12) being judged as inappropriate by investigators.

### Recruitment Strategies

We will recruit patients from the outpatient and inpatient populations of Neurology or Encephalology departments in seven centers (**Table 2**). Recruitment strategies will include publishing recruitment advertisements on social media (such as WeChat, QQ, etc., similar to Facebook), online publications, and at community centers. Patients who consent to participate will be examined and diagnosed by an associate chief physician and registered in an online allocation system after the inclusion criteria are confirmed and informed consent is signed.

### Randomization and Blinding

Central randomization will be performed using stratified and permuted blocks. According to the randomized clinical study program, with the help of the SAS 9.4 statistical software package PROC PLAN process, a stratified randomization method will be adopted to list the random coding table and generate the random arrangement of subjects’ treatment. Patients will be randomized in blocks of varying sizes within each center and stratified by centers.

All eligible participants will be randomly divided into the experimental group 1 (BHG group), experimental group 2 (NXTC group), experimental group 3 (YTG group), and placebo control group using a 1:2:1:1 ratio. According to the randomized clinical study program, the randomized code of the trial drug by statistical experts is the unique identification code of participants. Considering that the dosage forms include granules and capsules, a double-blind and double-simulation design will be adopted. All the participants, researchers, research assistants (conducting interviews with the participants), laboratory and inspection staff, and statisticians will be blinded to treatment assignment until the study is completed.

### Unblinding

The patient’s group allocation should be known in the event of adverse events (AEs) and can be obtained from the drug administrators when necessary. Researchers should contact the inspector and report the reasons for unblinding within 24 h. The precise cause of unblinding, the date of AEs, the treatment situation, and the results must be reported in the case report form (CRF) and signed by the administrator.

When the trial is completed, the data will be locked and cannot be changed after verification. Then the unblinding process will be conducted by the researchers, operated by the administrators, and the number of unblinding will be transferred to the sponsors.

### Intervention

The preparation and distribution of intervention drugs will be performed by Buchang Pharmaceutical Co., Ltd. The characteristics of the simulants in terms of packaging, color, shape, and flavor will be similar to those of BHG, NXTC, and YTG. After the treatment, drug packages will be returned to the investigators. Participants will receive the following interventions for 28 days.

***Experimental group 1***: capsule simulants (0.4 g/capsule, op, four capsules/time, thrice daily), BHG (5.5 g/package, op, one pack/time, thrice daily);

***Experimental group 2*:** NXTC (0.4 g/capsule, op, four capsules/time, thrice daily), inert granules (5.5 g/package, op, one pack/time, thrice daily);

***Experimental group 3*:** capsule simulants (0.4 g/capsule, op, four capsules/time, thrice daily), YTG (5.5 g/package, op, one pack/time, thrice daily);

***Placebo control group*:** capsule simulants (0.4 g/capsule, op, four capsules/time, thrice daily), inert granules (5.5 g/package, op, one pack/time, thrice daily).

All the participants will receive basic treatment in accordance with the “Guidelines for diagnosis and treatment of ischemic stroke in China” (Chinese Medical Association, 2015), and the drug and dosage will be formulated by the researchers according to the clinical situation. However, we should pay attention to the following guidelines: (1) only single antiplatelet drug therapy should be given; (2) except for experimental drugs and basic treatment, do not use any other TCM patent medicine or TCM therapy (including acupuncture) during the observation period; and (3) during the observation period, basic therapeutic drugs and other drugs or treatments that must be taken for complicated diseases should be listed in the CRF (e.g., drug name, usage, dosage, time of taking, etc.).

### Outcome Measures

#### Primary Outcome

The primary outcome will be the NIHSS score, which is commonly used to evaluate neurological recovery and quality of life in clinical practice (Yaghi et al., 2016). The NIHSS score will be measured at the end of the 28-day treatment period and compared with baseline, and changes will be compared between groups (**Table 3**).

**Table 3:** Measurement items and time line.

Visit	Screening	Intervention	Follow-up	
	V1	V2	V3	V4
Unit:day	-7-0	28 ± 4	90	180
Screening the subject	×			
Informed consent form	×			
Demographic information	×			
Inclusion/exclusion criteria	×			
Get the random number	×			
Previous medical history and allergy history	×			
Concomitant diseases and treatment history	×			
Connect to the Internet hospital “Doctor Tao” platform	×			
Head CT/MRI	×**^#^**			
Urine pregnancy test (HCG)	×^*^			
Vital signs^1^ and physical examination	×	×		
Blood routine^2^	×	×		
Urine routine	×	×		
Liver^3^ and kidney^4^ function	×	×		
Blood coagulation function^5^	×	×		
Fasting glucose	×	×		
12-lead electrocardiogram	×	×		
NIHSS	×	×	×	
TCM symptom score	×	×	×	
mRS score	×	×	×	
Barthel Index	×	×	×	
Biological effect indicators	×	×		
Metabolic and proteome data	×	×		
Pharmacokinetic index	×			
Drug distribution	×			
Recovery drug		×		
Record adverse events		×		×
Record drug combination		×		×
Compliance prediction		×		

^#^if imaging examination shows typical lesions after the onset and 4 weeks prior to the study can be avoided.

^*^Only women of child-bearing age were tested before the trial.

^1^Vital signs including the temperature, heart rate, respiration, blood pressure.

^2^Blood routine tests including red blood cells, hemoglobin, white blood cells, and platelets.

^3^Liver function tests including alanine and aspartate aminotransferases, alkaline phosphatase level, total bilirubin, and gamma-glutamyl transferase.

^4^Kidney function tests including creatinine and blood urea nitrogen.

^5^Blood coagulation function tests including prothrombin time, activated partial thromboplastin time, thrombin time, and fibrinogen.

#### Secondary Outcomes

The study has three secondary clinical efficacy end-points:

1. The modified Rankin scale (mRS) will be used to evaluate the subjects’ degree of disability. We will record the ratio in each group with mRS scores 0–5, and the proportion of subjects with mRS ≤1 in each group. Comparisons will be made on day 28 (immediately after treatment) and day 90 after IS onset, and statistical analysis will be conducted between groups.
2. The activity of daily living (ADL) scale will be measured with the Barthel Index (BI). The differences of BI scores (before and 28 days after treatment, and 90 days after onset) will be compared within and between the groups, and the proportion of participants with BI scores ≥90 will be recorded.
3. TCM symptom scores will be used to quantitatively score the symptoms. The differences in TCM symptom scores between groups will be compared for baseline and 28 days after treatment and 90 days after onset, and the differences between groups will be calculated. The comprehensive curative effect criteria are as follows: i) clinically resolved: symptoms and signs disappear or largely decrease, syndrome score decrease by ≥95%; ii) significant effect: symptoms and signs are significantly improved, syndrome score decrease by ≥70%; iii) effective: symptoms and signs are improved, syndrome score reduced by ≥30%; and iv) no effect: no significant improvement or even aggravation of symptoms and signs, <30% reduction of syndrome score (Jin et al., 2019). The formula for evaluation (nimodipine method) is:
  $$\text{comprehensive curative effect}=\frac{\text{integral before treatment}-\text{integral after treatment}}{\text{integral before treatment}}\times 100\%.$$

The number of total effective cases including clinically resolved cases, significant effect cases, and effective cases (i–iii).

#### Specific Indicators

Ten subjects per group will be randomly selected and blood and urine samples will be taken for specific indicator examination to explore the therapeutic biomarkers and pharmacodynamic material basis of PSC in treating patients with IS (QDBS syndrome). The study has three categories of specific indicators:

1. Biological indicators: including tissue-type plasminogen activator (t-pa), plasminogen activator inhibitor type 1 (PAI-1), D-dimer, interleukin-1 (IL-1), IL-6, tumor necrosis factor-alpha (TNF-α), high-sensitivity C-reactive protein (hs-CRP), matrix metallo proteinase-9 (MMP-9), platelet aggregation rate, endothelin, nitric oxide (NO), adenosine triphosphate (ATP), superoxide dismutase (SOD), glutathione peroxidase (GSH-PX), malondialdehyde (MDA), and peroxidase (POD).
2. Metabolomic indicators (blood and urine samples): gas chromatography–mass spectrometry (GC–MS), liquid chromatography-mass spectrometry (LC–MS), and other technologies will be used to reveal the chemical and biological fingerprints, screen effective components, and explore the possible metabolic pathways and target molecules of BHG, NXTC, and YTG for treating IS with QDBS.
3. Proteomics (blood samples): two-way fluorescence differential gel electrophoresis (2D-DIGE) will be used to assess the proteome of each group before and after intervention. Matrix-assisted laser desorption/ionization time-of-flight/time-of-flight mass spectrometry (MALDI TOF/TOF MS) techniques will be used to identify the differentially expressed proteins. Bioinformatics analyses will be performed to assess the biological functions of differentially expressed proteins, and STRING and Ingenuity Pathway Analysis will be used to establish the protein network affected by BHG, NXTC, and YTG.

Blood sample collection requirements: subjects fast 10 h before each collection, 5 ml blood is drawn and centrifuged, and the serum stored in Eppendorf tubes at −70 °C. Urine sample collection requirements: subjects fast 10 h before taking medicine (allow to drink water), fast and water prohibit 1 h before urine collection to 2 h after taking medicine, ration water at 200 mL/h for 2–8 h after taking medicine, and consume a low-fat meal 4 h after taking medicine. Urine samples will be collected at baseline and after treatment (28 days) and stored in Eppendorf tubes at −70 °C.

To guarantee the consistency of the laboratory tests, the proteomics and metabolomics experiments will be carried out in a centralized lab. This pilot study is designed to precisely identify biomarkers of IS with QDBS syndrome and the bioactive constituents of BHG, NXTC, and YTG related to PSC.

### Safety and AE Monitoring

In this study, safety will be monitored by clinical research associates including AEs, serious AEs, withdrawals or treatment modification due to AEs, vital signs and physical examinations, routine blood and urine tests, liver and kidney function tests, blood coagulation function test, fasting glucose test, 12-lead electrocardiogram, and concomitant medications for management of AEs. The severity of AEs will be graded using CTCAE version 3.0. The indexes and time points for data collection are shown in **Table 3**.

### Sample Size Estimates

This trial is a pilot study. Based on the superiority clinical trial hypothesis test sample size estimation (Sakpal, 2010) and data from preliminary observations and case-control studies (Sun, 2010; Wan et al., 2015; Cheng et al., 2019), we assumed that over a 28-day period, the minimum difference of NIHSS score between the experimental and placebo groups would be 0.95. With a 2-year enrollment period and 180-day follow-up period, taking into account a 20% dropout rate, we predicted that a total of 160 participants would achieve 80% power and a two-sided 5% significance level. Therefore, according to the ratio of 1:2:1:1, we will recruit 64 patients in experimental group 2 and 32 patients each for experimental groups 1 and 3, and the placebo control group.

### Statistical Analysis Plan

Statistical analysis will be performed using SPSS version 15.0 for Windows (Chicago, IL, USA). Professional statisticians who are independent of all the other processes of the study will perform the statistical analyses. Consistent with the CONSORT statement and intention to treat principle, the last observation carried forward method will be used for missing values. Cases in the per protocol set (PPS) are those who thoroughly adhere to the protocol without absence of baseline characteristics. Analyses of the primary outcome and curative effect will be carried out using a full analysis set approach and PPS approach. The safety analysis set will include all randomized patients who have completed at least one study visit. Participating centers will be required to count the number of participants in each center and list those who are removed from the PPS. For quantitative data, we will calculate the mean, standard deviation, median, minimum, maximum, lower quartile (Q_1_), upper quartile (Q_3_); and for qualitative data, we will describe numbers and percentages. Inter-group comparisons will be analyzed by appropriate methods: independent t-tests (homogeneity of variance, normal distribution) or Wilcoxon rank-sum test for quantitative data, chi-square test or Fisher’s exact test (if chi-square test is not applicable) for qualitative data, and Wilcoxon rank-sum test or Cochran-Mantel–Haenszel test for ranked data. *P* <0.05 will be considered statistically significant.

### Quality Control of the Intervention

To ensure the quality of this trial, a multicenter trial coordination committee and general director will be tasked with solving related problems. The coordination committee includes the leaders of each center and the head sponsor who will all complete pre-clinical training. All staff including the operators, researchers, physicians, data collectors, and analyzers will fully understand the purpose and content of the trial. This trial will be inspected by the China Food and Drug Administration (CFDA), sponsor, and clinical research organization throughout the process.

The sponsor will appoint inspectors to ensure the rights and interests of the participants and the accuracy and integrity of data. They shall also supervise the implementation, management norms, and relevant regulations of this trial and provide regular on-site supervision. In the monitoring process, if the CRF table does not conform to the protocol, the inspector has the right to propose suggestions for modification. If the CRF table is not standardized (e.g., input errors), researchers are responsible for following the original data for correction. Furthermore, pre-clinical trial training will be provided to ensure that researchers fully understand the protocol and the specific contents of each index. Objective indexes should be tested according to the time points and methods specified in the protocol, and the AEs or unexpected toxic and side effects should be observed and followed up.

## Discussion

The high disability rate of stroke brings a serious burden to patients, their families, and society; promoting neurological function recovery is critical to improve the prognosis of patients with stroke. Integrative medicine that combines TCM with western medicine has emerged as an optimized method to improve outcomes in patients with IS. As a supplementary and complementary medicine strategy, TCM is attracting increasing attention. It uses a theoretical system of treatment based on syndrome differentiation, and its good curative effect need to be evaluated with modern methods. However, the investigations based on the patient’s symptoms and signs are affected by the subjectivity of doctors, and some symptoms are relatively hidden, making them difficult detect and precisely treat. In the age of “precision medicine,” we urgently need to improve the relevant degree of PSC.

BHG is widely used in IS of QDBS syndrome, and prepared by Shaanxi Buchang Pharmaceutical Co. Ltd. Preparation methods of BHG: according to the original prescription and decoction method of BHD of Yilin Gaicuo, all herbs were purchased and identified by the pharmacy department, prepared in proportion, soaked, decocted twice, concentrated, spray dried, then packaged into 5.5 g/package for use. NXTC (national medicine permission number: Z20025001), produced by Shaanxi Buchang Pharmaceutical Co. Ltd., obtained drug production approval in 1993, was awarded a National Chinese Medicine Protection Certificate in 2014, and is included in the National Basic Drug List (2012 edition) and the Chinese Pharmacopoeia (2015 edition). Over the past two decades, NXTC has been used to treat more than 100 million patients, and more than 1,000 clinical or basic research papers were published (‘Chinese Experts Consensus on Clinical Application of Naoxintong Capsule’ compilation team, 2017; Liu and Fu, 2017). A systematic review and meta-analysis of a total of 1,141 cases included in randomized trials were performed to assess the efficacy of NXTC as adjuvant therapy for IS with QDBS syndrome. The authors reported that NXTC could significantly improve NIHSS score, improve blood lipid parameters, and attenuate TCM symptoms (e.g., hypoesthesia, white complexion, shortness of breath, fatigue, dark tongue, and irregular pulse etc.) (Qiuer et al., 2018; Zhu et al., 2019). An RCT assigned 310 cerebral infarction patients (QDBS syndrome) within 72 h after the event to a treatment group (192 cases, given NXTC four capsules/time, thrice daily on the basis of routine treatment) and a control group (118 cases, given conventional treatment) who were treated for 15 days. The total effective rate of the treatment group was significantly higher than that of the control group (94.27% *vs.* 66.95%, *P* <0.01) (Sun, 2010; Liu and Fu, 2017). YTG is a modern Chinese medicinal compound developed by professor Wan Hai-tong who successfully obtained a patent and approval from the CFDA (approval No.: 2003L00206). YTG has effects of nourishing *Qi* and *Yin* and removing blood stasis, making it is suitable for the treatment of QDBS syndrome in IS patients. From 2008 to 2013, phases II and III clinical trials of YTG were completed and have passed the CFDA expert review. The results showed that YTG is safe and effective for patients with IS (QDBS syndrome) in the convalescence period and can promote neurological recovery, improve cognitive function, and enhance abilities in daily life (Wan et al., 2015; Wang et al., 2017; Wang et al., 2019).

The study design will include IS cases with QDBS syndrome to explore the correlation degree of PSC by invigorating *Qi* and activating blood circulation prescriptions application. The NIHSS score was selected as the primary outcome, and TCM syndrome score was selected as the secondary outcome, to reflect both the advantages and characteristics of TCM and the objective efficacy evaluation of western medicine (Wang et al., 2013). By employing proteomics and metabolomics techniques, we can explore the biological basis of PSC, and improve the treatment of IS by providing an objective basis for precise treatment. This protocol was designed according to the SPIRIT 2013 statement and the SPIRIT 2013 explanation and elaboration (Chan et al., 2013b).

However, there are several limitations in this study, such as the lack of assessment of the long-term effects of invigorating *Qi* and activating blood circulation prescriptions on primary outcome measures. The treatment period was only 28 days with 180 days of follow-up, which is relatively short. Due to the limited time frame, the potential roles of prescriptions in reducing overall mortality and major vascular events over the long term remain uncertain. Future RCTs should include longer follow-up periods.

## Ethics Statement

The studies involving human participants were reviewed and approved by This protocol was approved by the Research Ethics Committee of the First Affiliated Hospital of Zhejiang Chinese Medical University (ID: 2017-Y-004-02). The patients/participants provided their written informed consent to participate in this study.

## Author Contributions

HW and JY conceived and designed this study. YW and LZ wrote the manuscript with contributions from all authors. YP, SH, WF, BX, LD, QH, CL, LY, and HZ refined the protocol. All authors contributed to the article and approved the submitted version.

## Funding

This project is supported by the National Natural Science Foundation of China (No.81630105), the National Key R&D Program of China (2019YFC1708600, 2019YFC1708603), and the National Natural Science Foundation of China (No.81973560).

## Conflict of Interest

The authors declare that the research was conducted in the absence of any commercial or financial relationships that could be construed as a potential conflict of interest.
