# Supplementary material for: Investigation of Invigorating Qi and Activating Blood Circulation Prescriptions in Treating Qi Deficiency and Blood Stasis Syndrome of Ischemic Stroke Patients: Study Protocol for a Randomized Controlled Trial
Source: Front Pharmacol. 2020 Jun 17;11:892. doi: 10.3389/fphar.2020.00892 (PMC7311665; doi:10.3389/fphar.2020.00892)
Supplement: Supplementary file 1 [file DataSheet_1.pdf]

Supplementary Material

1 Supplementary Figures

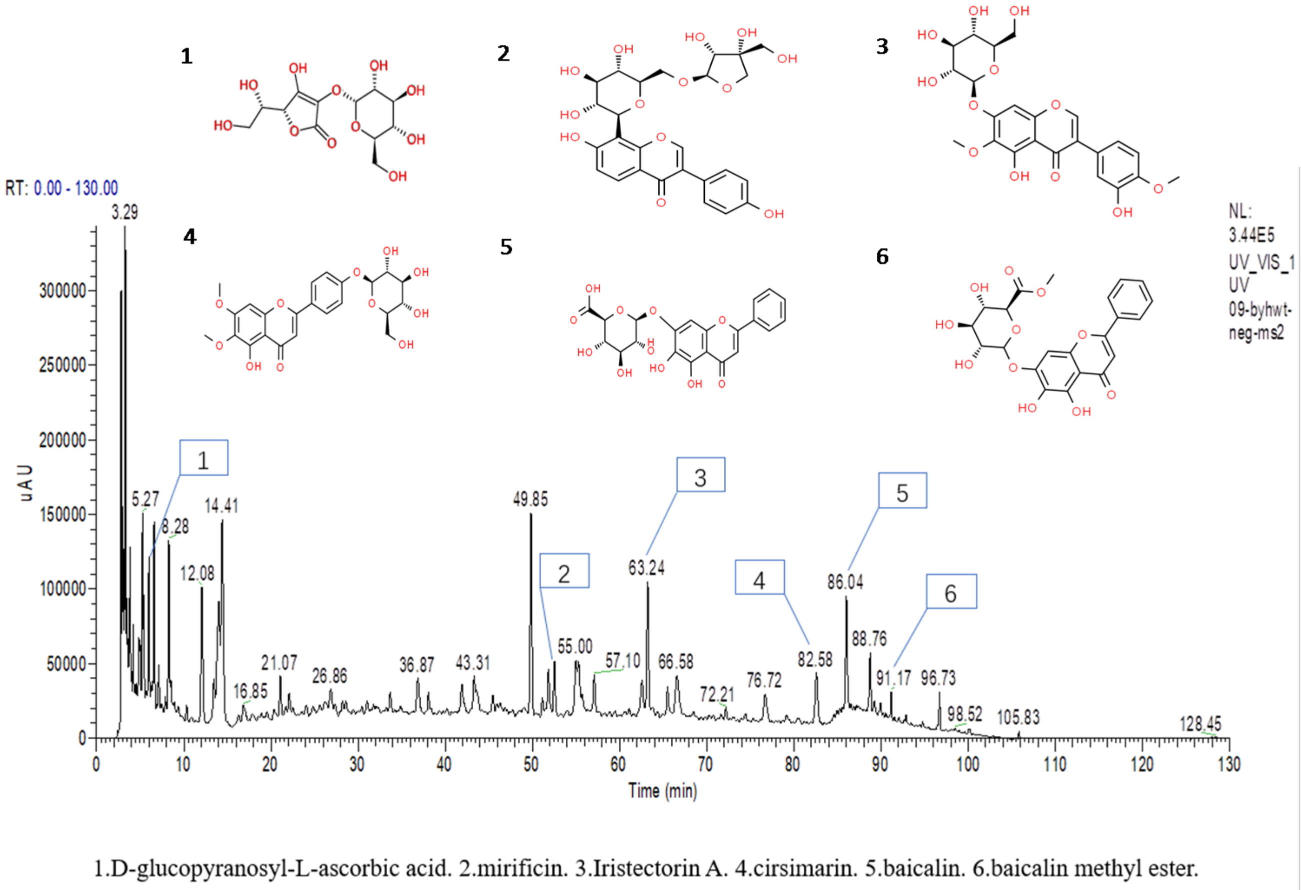

Figure 1. HPLC chromatogram of BHG. 1-D-glucopyranosyl-L-ascorbic acid, 2-mirificin, 3-iristectorin A, 4-cirsimarin, 5-baicalin, 6-baicalin methyl ester. HPLC conditions: column, Sunfire C18 (250 mm×4.6 mm i.d., 5 µm, Waters); column temperature, 25 °C; mobile phase, acetonitrile and 0.5% formic acid in water at the gradient: acetonitrile, 0–10 min, 2%, 10–35 min, 2-10%, 35–50 min, 10–15%, 50-80 min,15-25%,80-100 min, 25-60%, 100-110 min, 60-90%, 110-120 min, 90%; flow rate, 0.8 mL/min; detection, 254 nm UV.

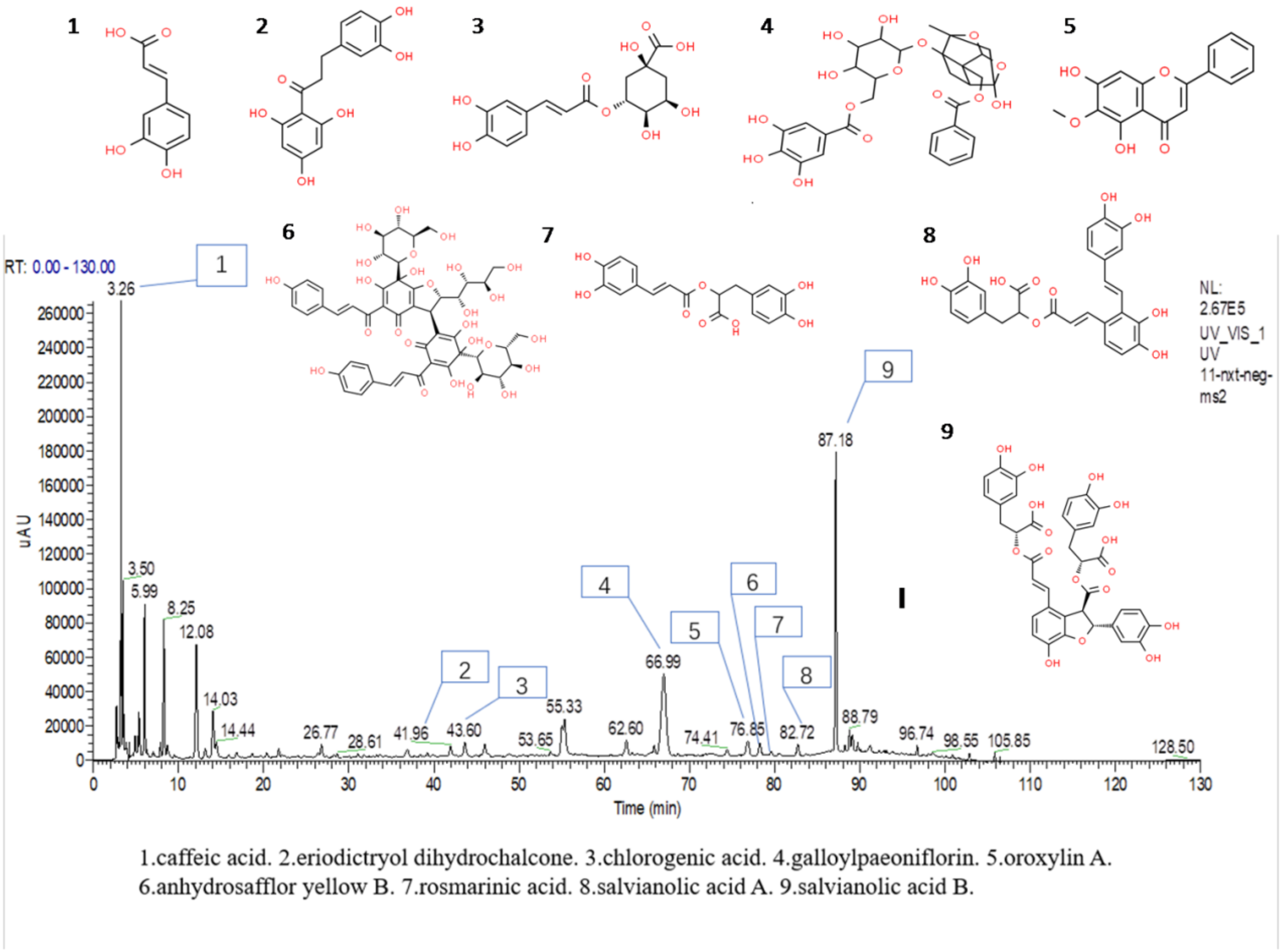

Figure 2. HPLC chromatogram of NXTG. 1-caffeic acid, 2-eriodictryol dihydrochalcone, 3-chlorogenic acid, 4-galloylpaeoniflorin, 5-oroxylin A, 6-anhydrosafflor yellow B, 7-rosmarinic acid, 8-salvianolic acid A, 9-salvianolic acid B. HPLC conditions: column, Sunfire C18 (250 mm×4.6 mm i.d., 5 µm, Waters); column temperature, 25 °C; mobile phase, acetonitrile and 0.5% formic acid in water at the gradient: acetonitrile, 0–10 min, 2%, 10–35 min, 2-10%, 35–50 min, 10–15%, 50-80 min,15-25%,80-100 min, 25-60%, 100-110 min, 60-90%, 110-120 min, 90%; flow rate, 0.8 mL/min; detection, 254 nm UV.

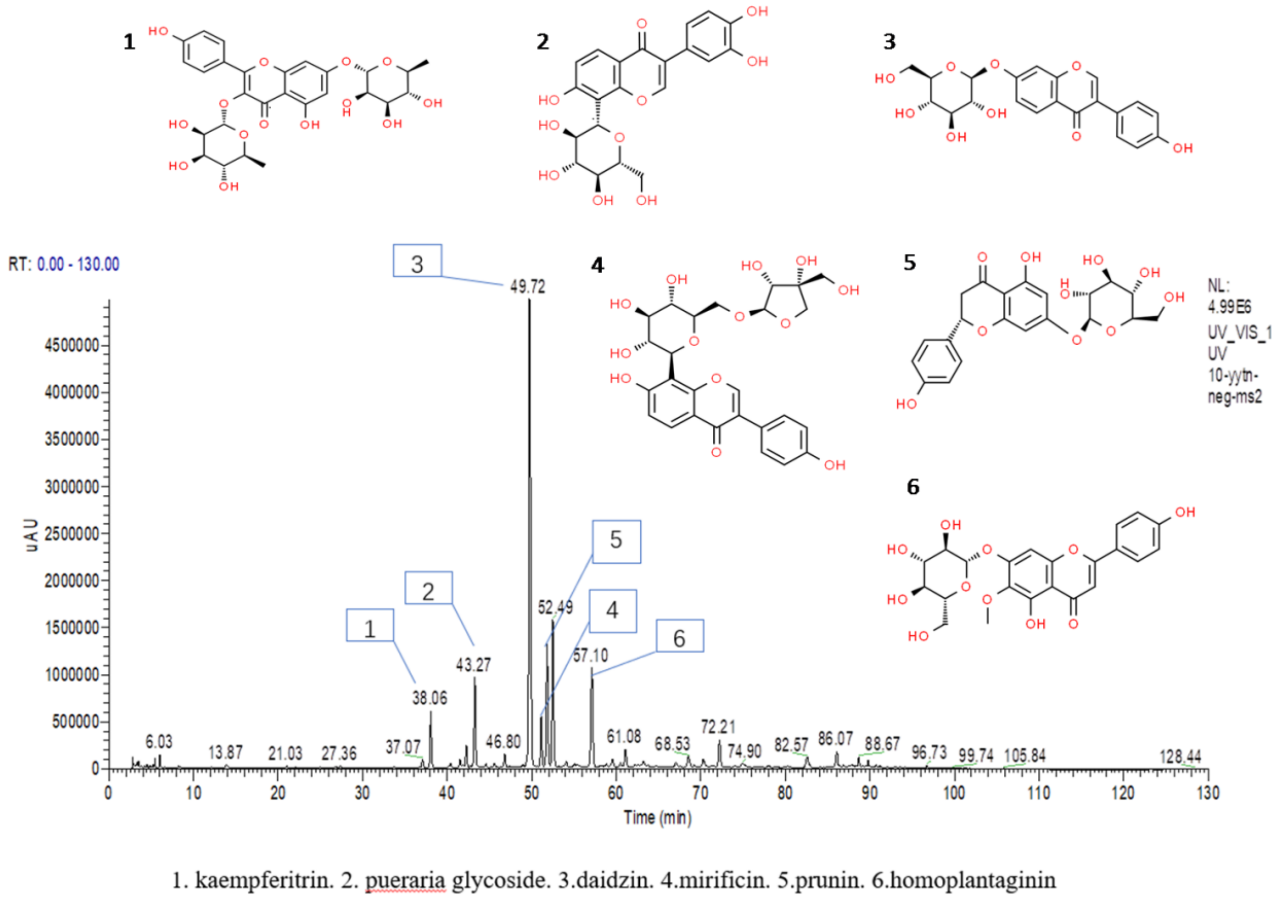

Figure 3. HPLC chromatogram of YTG. 1-kaempferitrin, 2-pueraria glycoside, 3-daidzin, 4-mirificin, 5-prunin. 6-homoplantaginin. HPLC conditions: column, Sunfire C18 (250 mm×4.6 mm i.d., 5 µm, Waters); column temperature, 25 ℃; mobile phase, acetonitrile and 0.5% formic acid in water at the gradient: acetonitrile, 0–10 min, 2%, 10–35 min, 2-10%, 35–50 min, 10–15%, 50-80 min,15-25%,80-100 min, 25-60%, 100-110 min, 60-90%, 110-120 min, 90%; flow rate, 0.8 mL/min; detection, 254 nm UV.
